# A Clinical Review of Some Cases in Private Practice

**Published:** 1911-02

**Authors:** Thos. E. Mitchell

**Affiliations:** Columbus, Ga.


### A CLINICAL REVIEW OF SOME CASES IN PRIVATE PRACTICE.

By Thos. E. Mitchell, M. D., of Columbus, Ga.

While I do not appear before you having found something new under the sun, I nevertheless believe that the cases which I

propose to report bear each so much of merit as to form a justifiable excuse for this paper.

Case 1.—Annie P., female, aged 10 years, was brought from a neighboring village in October, 1897, with the following history, as learned from her father.

Of his five children, three had suffered from very sore throats. When convalescence was well established they went to spend some days at the home of a relative where there were other children, some of whom developed sore throats a few days subsequent to the arrival of their guests.

During the second week of convalescence the child whose case is being reported, became very nervous, and, "as she was not doing well," she was sent home, where she rapidly developed into the condition in which I found her a few days later when I saw her for the first time, namely, extremely nervous, weak and irregular heart, unsteady gait, loss of tendon reflexes, extreme convergent strabismus, paralysis of the soft palate both motor and sensory, with regurgitation of liquids through the nose during deglutition.

The family physician, who insisted that the recent pharyngitis was only a "stubborn sore throat," diagnosed the present condition as "spinal disease," and had prescribed, among other things, an "Allcock's Porous Plaster," which was pasted to the little sufferer's back at the time I first saw her.

Thus armed with a complete history of the case, I had no difficulty in diagnosing diphtheritic paralysis, and, barring a possible fatal cardiac involvement, was able to make an extremely favorable prognosis. Generous tonics, a nourishing dietary, together with rest, effected a complete cure in a very few weeks.

Case 2.—In the fall of 1897 I was called to see L. F., a female mulatto, aged 34, whom I found in bed, temperature 102, and complaining of intolerable pains above and in front of the left ear, which region was exquisitely sensitive to the touch, though not perceptibly swollen.

She was unable to breathe through the left side of her nose, which was a little swollen on the outside and exceedingly painful to the touch. For this pain she had been taking on her own account from one to two grains of morphine a day.

Two other physicians had seen the case two or three times each at intervals previous to my being called, though she had not been regularly under the care of any one.

Her health began to fail about eight months prior to my first visit, during which time her weight, which for some years had been 175 pounds, had gradually fallen to 115 pounds.

Amenorrhoea had existed for five months. She was the mother of only one child, which was ten years of age, and in good heath.

Following a well-known rule of whist which demands a trump lead when in doubt, I prescribed 1-16 of a grain of corrosive sublimate three times a day, in connection with the iodide of potassium in increasing doses until the point of toleration had been reached.

My doubtful diagnosis of specific infection did not long remain so, because in no instance have I seen symptoms yield more readily. Indeed, it was not unlike the melting of snow-drops before a morning sun.

In a very few days the pain was practically a thing of the past, and in a remarkably short while the occluded nasal passage was again patent without local treatment.

Within three months from the time treatment was begun the recorded loss of more than fifty pounds in weight had been regained, while the patient to all intents and purposes was in perfect health with each emunctory organ doing its full physiological duty.

Case 3.—In May, 1901, Henry W., colored, aged 24, came for consultation and gave the following history:

Four years previously, by the accidental explosion in his hands of an old-style pistol, the right eye was injured.

Although no physician was consulted at the time, the pain and irritating symptoms incident to the injury soon subsided and the eye became comfortable, though there was a spot on the cornea, near the pupil.

The eye remained in this condition, giving its possessor no pain and but little inconvenience, until a short while prior to the first time I saw him.

Upon examination I found the conditions as follows:

Toward the temporal side of the right cornea there was an infiltrated area accompanied by pain, photophobia, lachrymation and circumcorneal congestion.

By focal illumination I was able to see what subsequently proved to be a large grain of powder deeply imbedded in the inflamed tissue and apparently just ready to drop into the anterior chamber.

Feeling sure that I could not safely remove the foreign body from in front through the tract of entrance, I made an incision in the cornea at the temporal side of its transparent margin with a triangular keratome, not unlike such an incision as would be made for an ordinary iridectomy for optical purposes.

Through the opening thus made I was enabled to remove the offending body with iris forceps.

The recovery was rapid, uneventful and perfect, there being but little remaining opacity, which, fortunately, was well outside the central field of vision.

To my mind this case is interesting in that the foreign body was driven into and nearly through the cornea where it remained, encysted as it were, without symptoms of irritation for four or more years.

